# Comparing the Effectiveness of Computer-Based, Manual-based, and Combined Cognitive Rehabilitation on Cognitive Functions in Relapsing-Remitting Multiple Sclerosis Patients

**DOI:** 10.32598/bcn.9.10.430

**Published:** 2020-01-01

**Authors:** Mehri Rahmani, Isaac Rahimian Boogar, Siavash Talepasand, Mostafa Nokani

**Affiliations:** 1. Department of Clinical Psychology, Faculty of Psychology and Educational Sciences, Semnan University, Mahdishahr, Semnan, Iran.; 2. Department of Clinical Psychology, Arak University of Medical Sciences, Arak, Iran.

**Keywords:** Cognitive rehabilitation, Executive functions, Working memory, Selective attention, Multiple sclerosis (MS)

## Abstract

**Introduction:**

This study aimed to compare the effectiveness of computer-based, manual-based, and combined cognitive rehabilitation to improve cognitive functions among patients with Relapsing-Remitting Multiple Sclerosis (RRMS).

**Methods:**

This was a quasi-experimental study with a pre-test-post-test, and a 2-month follow-up, as well as a control group design. Sixty female patients with RRMS were selected by convenience sampling technique. Then they were randomly assigned into three experimental groups (computer-based, manual-based, and combined cognitive rehabilitation, each group including 12 patients), a placebo group (12 patients), and a control group (12 patients). The interventions were conducted in 21 sessions for the experimental groups for 5 months. The placebo group received physical rehabilitation intervention, and the control group received no intervention. The study participants were assessed by Psychiatric-Neurological Profile, Mini-Mental State Examination, Kurtzke Expanded Disability Status Scale, Stroop Color and Word Test, Wisconsin Card Sorting Test, and Paced Auditory Serial Addition Test. The obtained data were analyzed by repeated-measures multivariate analysis of variance in SPSS.

**Results:**

The effect of group factor was not significant (η^2^=0.129), but the effect of time (η^2^=0.884) and interaction effect of time and group (η^2^=0.295) were statistically significant (P<0.05). There was no significant difference between the effects of all three rehabilitation interventions (P>0.05). In the post-test totally and in Follow-up partially, all comparisons among three experimental groups with control and placebo groups were statistically significant (P<0.05).

**Conclusion:**

Cognitive rehabilitation was effective in improving cognitive functions in patients with RRMS. Thus, these interventions are recommended for application, along with other treatment protocols to treat RRMS in clinical settings.

## Highlights

- The importance of the cognitive rehabilitation to improve cognitive functions among patients with Relapsing-Remitting Multiple Sclerosis.
- Women are more likely than men (2 to 1 ratio) apt to MS between the ages of 30 and 35 years.
- The interventions are recommended for application, along with other treatment protocols to treat RRMS in clinical settings.

## Plain Language Summary

MS is an inflammatory disease of the Central Nervous System (CNS) that causes the demyelination of nerve cells. Working memory is prone to major disruptions in this disease.In this study, the subjects were assigned into three experimental groups (computer-based, manual-based, and combined cognitive rehabilitation, each group including 12 patients), a placebo group (12 patients), and a control group (12 patients). There was no significant difference between the effects of all three rehabilitation interventions. Cognitive rehabilitation, as an attempt to restore lost cognitive capacities by training and providing targeted incentives for accepting, aims to improve individuals’ performance in conducting activities; it is a treatment mainly aimed to improve deficiencies in cognitive performance, such as memory, executive functions, social perception, attention, problem-solving, and judgment as well as disorders in cognitive, mental, motor, and behavioral skills in patients.

## 1. Introduction

Multiple Sclerosis (MS) is an inflammatory disease of the Central Nervous System (CNS) that causes the demyelination of nerve cells and destroys oligodendrocytes, neurons, and axons (Huseby, et al 2015). Studies have suggested that women are more likely than men (2 to 1 ratio) apt to MS between the ages of 30 and 35 years (Goodin, 2016). Although the primary cause of the disease remains undiscovered, the main hypothesis is based on the activity of regulatory T cells in an autoimmune process (Bittner, Ruck, Wiendl, Grauer, & Meuth, 2017). Additionally, studies have revealed that genetic, immunological, and environmental factors are involved in the pathogenesis of this multi-factorial disease. The most prevalent demonstrations of the disease include paresthesia or numbness, diplopia, weakness, fatigue, and dizziness (Zahoor, Asimi, Haq, & Wani, 2017). The 4 pathological courses that have been recognized in MS include clinically isolated syndrome, Relapsing-Remitting MS (RRMS), secondary progressive MS, and primary progressive MS (Huseby, et al 2015; Goodin, 2016; Zahoor et al., 2017). RRMS is the most prevalent MS, which included more than 80% of the cases. In the early stages of the disease, attacks are unpredictable, and symptoms suddenly appear at any time. The patient’s symptoms will continue a few days or a few weeks and will disappear again. It seems that there is no MS disease progression between attacks, and it is possible that the patients be asymptomatic for numerous years (Burks, Bigley, & Hill, 2009). The present study examined the cognitive problems in RRMS that patients might experience. Cognitive impairments in MS were properly outlined by a French neurologist, Dr. Jean Martin Charcot (1825–1893), who described the clinical features of this disease in 1877 (Johnen et al., 2017). Based on the neuropsychological tests, most MS patients suffer from cognitive impairment (Ferreira, 2010). Besides, the onset speed of this condition is relatively high and can affect personal and professional life (Hämäläinen & Rosti-Otajärvi, 2016). Cognitive impairments are very diverse among MS patients. In this regard, working memory, information processing speed, executive functions, and attention are the most common at-risk functions in MS (Fuso, Callegaro, Pompéia, & Bueno, 2010).

Working memory is prone to major disruptions in this disease, i.e. mild or inconsiderable in the early stages of MS (Vacchi et al., 2017). Working memory is defined as brain function that temporarily stores information and manages them to conduct activities (Sohlberg & Mateer, 2017; Costa, Genova, DeLuca, & Chiaravalloti, 2017). Ferreira, in his study about working memory among MS patients and comparing them with healthy subjects, found that damage in the frontal lobe could reduce the performance of working memory (Ferreira, 2010).

The declination in the information processing speed is a key issue in MS, which could be present in 20%–30% of patients. Information processing efficacy is the brain’s ability to maintain and manipulate data in the shortest time. Processing speed deficits, which are observed even in the most basic activities of MS patients, are related to the slow axonal transport due to demyelination. The slowing of information processing may affect an individual’s ability to complete tasks and impact heavy tasks (Covey, Zivadinov, Shucard, & Shucard, 2011). Executive functions are cognitive abilities, including planning, organizing, reasoning, and abstract conceptualization that are necessary for coping with environmental changes (Ferreira, 2010; Lincoln et al., 2015). Patients with MS, compared with the control group, are faced with some task performance problems related to executive functions, like card-sorting assignments (Lincoln et al., 2015).

Patients with MS also have problems with selective attention. Selective attention is combining different information, which has an important function for the activities of executive systems (Klein, Drummond, Mhizha-Murira, Mansford, & das Nair, 2017). The mechanisms of selective attention help us choose the required information among extensive data. Selective attention is an aspect of the inhibitory control system. Attention distraction, failure in careful concentration on special stimulus, and discovering complicated stimuli with affective methods are other difficulties mostly observed among MS patients (Llufriu et al., 2016). Many MS patients with deficits in executive functions have major difficulties in complex functions, like selective attention (Nejati, Shahidi, & Helmi, 2016; Campbell, Langdon, Cercignani, & Rashid, 2016).

The assessment and intervention provision for cognitive functions is essential in patients with MS. However, it is illogical to rely only on routine consulting psychology. Obvious and visible signs usually cover cognitive symptoms (e.g. motor, sensory, and balance difficulties) that may cause latent cognitive symptoms. Besides, They might generate convert emotional complications, such as depression, fatigue, and pain, which often remain unrecognized and untreated by clinicians through tailored therapy.

Cognitive rehabilitation, as an attempt to restore lost cognitive capacities by training and providing targeted incentives for accepting, aims to improve individuals’ performance in conducting activities (Kellay & O’Sullivan, 2015). Cognitive rehabilitation is a treatment mainly aimed to improve deficiencies in cognitive performance, such as memory, executive functions, social perception, attention, problem-solving, and judgment as well as disorders in cognitive, mental, motor, and behavioral skills in patients (Bonavita et al., 2015; Amato et al., 2014; Hancock, Bruce, Bruce, & Lynch, 2015). The psychoneurological intervention studies introduce two cognitive rehabilitation protocols and compare their effectiveness for improving cognitive performance in MS patients. No survey was conducted in this field, and in this viewpoint, it considered as applied research. The present research aimed to compare the effectiveness of computer-based, manual-based, and combined cognitive rehabilitation in the cognitive function improvement of female patients with RRMS. We also explored the difference between these rehabilitation methods in the cognitive function improvement of these patients.

## 2. Methods

The study was a quasi-experimental with a pre-test-post-test, 2-months follow-up, a placebo, and a control group design. The population consisted of all patients with RRMS in Arak City, Iran, from July 2016 to March 2017. Sixty female RRMS patients were selected by convenience sampling method and then randomly assigned into 5 groups of 12 subjects, including three experimental groups (computer-based, manual-based, mixed cognitive rehabilitation training), one placebo group receiving physical rehabilitation intervention, and one control group that received no intervention.

The study inclusion criteria were the range of 18–45 years, Expanded Disability Status Scale (EDSS) score of ≤3.5, Mini-Mental State Examination (MMSE) score of 10–20, and at least a moderate literacy level. In addition, the exclusion criteria included pregnancy during the study or decision to pregnancy at the beginning of the study, the lack of regular medical checkup or follow-up treatment, and MS attack in the one past month and during the study, psychotic/major psychopathological comorbidities, major medical comorbidities, and hearing or speaking difficulties.

To control the confounding factors, the medication type, dose, and duration of its consumption were controlled in terms of the medication type and matched by the conditions of drug consumption for all patients. In addition, the disease severity on the onset and during the study was controlled by a neurologist. All three cognitive rehabilitation interventions were implemented by three MSc in clinical psychology that had been trained and supervised by the researcher in Arak Payam Noor University Counseling Center. Physical rehabilitation intervention in the placebo group was conducted by a sports and health specialist in Arak City.

The Psychiatric-Neurological Profile (PNP), MMSE, and EDSS were conducted at the initial screening for recording individual history characteristics and to consider the inclusion/exclusion criteria. The PNP included a neurologist diagnosis, signs, symptoms, physical and clinical examinations, and the results of para-clinical tests, like Magnetic Resonance Imaging (MRI) for MS diagnosis. The MMSE was developed by Folstein, Folstein, and McHuge (1975) to assess cognitive functions, such as orientation, language, attention and concentration, calculation, recall, and perception. This test’s reliability is acceptable. The 24-hour test-retest reliability of it was measured as 0.89 among patients with dementia. In the study by Folstein et al., (1975), the 4-week interval test-retest reliability of it was gained as 0.99 in patients with dementia. The EDSS, developed by Kurtzke (1983), quantifies disability in MS patients. The EDSS is an 8-option scale, based on the illness stages. Moreover, its scores indicate an objective measurement of neurological disability levels in MS patients. In this system, scores vary from 0 for healthy operations to 10 for death due to MS. This scale evaluates 8 functional systems; pyramidal, cerebellar, brain stem, sensory, bowel, and bladder, visual, cerebral (e.g. thinking), and other Kurtzke (1983).

Besides, the Stroop Color and Word Test (SCWT), the Wisconsin Card Sorting Test (WCST), and the Paced Auditory Serial Addition Test (PASAT) were applied as neuropsychological measurements to collect the outcome data on cognitive functions at the pre-test, post-test, and follow-up assessments. The SCWT was originally proposed by Stroop (1935), as a classical test for assessing frontal lobe, selective attention, and executive control. Generally, in the Stroop effect phenomenon, the impact of the stimulus on human attention and the ability to switch attention from one dimension to another is examined.

Although the test consists of 4 stages in its original form, in the present computer-based study, it was divided into 3 stages, as follows: a. The first level, i.e. the level of coordinated trials, the names of 4 main colors with black color appear in the center of computer screen, and patient should push one of the blue, red, yellow, or green keys according to their names as quickly as possible; b. At the second level, the names of 4 colors with their colors appear on the computer screen, and patient should push the keys according to their colors as quickly as possible; and c. The third step examines the level of inconsistent or interference trails. Through these levels, the names and colors appear with different colors, and the respondent should push the keys as quickly as possible. According to Stroop (1935), the index of the Stroop test contains accuracy (the number of correct answers) and speed (the average time of correct responses against stimulus in milliseconds).

The WCST was designed by David A. Grant and Esta A. Berg in 1948 for assessing the problem-solving and decision-making skills. of the WCST is among the main indexes for frontal lobe activity. It is currently applied to evaluate transfer-response amounts, i.e. one component of the executive functions. Electrophysiological research and imagery functional of the brain revealed a relationship between the frontal lobe’s activity and the WCST results (Grant & Berg, 1948). The test’s validity to assess cognitive impairment and its reliability is high (Kohli & Kaur, 2006). The Paced Auditory Serial Addition Test (PASAT) was developed by Gronwall in 1977, then adapted by Rao et al. in 1989 to assess working memory and information processing speed in MS patients (Tombaugh, 2006). Studies have reported that the results of memory tests are significantly correlated with each other in MS patients (Tombaugh, 2006). Standard PASAT test consists of 61 numbers, which broadcast auditory surveys at 3-second intervals. After hearing each number, respondents are requested to keep it in their memory and add them to the next number that they hear and immediately announce the sum (Negels et al., 2005).

The present study interventions were implemented using the Pars Cognitive Rehabilitation Package and Captain’s Log Computerized Cognitive Training System. Pars Cognitive Rehabilitation Package consists of programs for Neurocognitive Joyful Attentive Training Intervention, which was developed by Nejati et al. (2016) in the Cognitive Neuroscience Center at Shahid Beheshti University. Besides, it includes exercises for improving attention and working memory. This is a series of a pen-paper program for improving executive functions and consists of a hierarchically-organized group of assignments that reinforce different aspects of executive functions. The program is based on a hierarchical model (Nejati et al., 2016) . In this model, underlying cognitive and molecular processes are important. Thus, initially, the concepts of cognitive domains, such as conceptualizing, planning, and memory, are aimed at treatment through frequent exercises. Then, skills training and higher-level functions are targeted by the intervention.

Through this program, assignments are designed in a hierarchy, and their difficulties increase based on the user’s response beyond sessions. Assignments are organized based on various functions of attention, working memory, and inhibition. These assignments can be repeated until the patient reaches the desired level. The decision to develop the program is made based on the patient’s efficiency. Through this program, the therapist must be present to improve the assignment level (Sohlberg, Mateer, 2017). Captain’s Log Computerized Cognitive Training System is an excellent training kit for improving high-level cognitive functions and processes.

The first version of this instrument was developed in 2001 by the Brain Train Company. It has 2000 programs and different assignments at different levels to improve cognitive functions, including various precision and concentration (selective precision, focused precision, continuous precision, dissociated precision and shifting attention), working memory, instant memory, and short-term visual and audio memory, visual and auditory processing speed, auditory and visual perception, sensorimotor coordination, improve hand-eye coordination, visual processing, and micro-motion control, problem-solving skills, executive functions, and the speed of response.

The study participants in all groups were tested at the pre-test phase before initiating the study. The study was conducted over 21 sessions of cognitive rehabilitation for the intervention groups (5 months). Finally, all study groups were assessed at post-test phase. In addition, two months after the intervention, all study groups were evaluated at the follow-up phase.

Manual Cognitive Rehabilitation Protocol was designed based on the cognitive rehabilitation guideline by Kellay and O”Sullivan (Kellay & O’Sullivan, 2015), Pars Cognitive Rehabilitation Package by Nejati, Shahidi and Helmi (2016) as Neurocognitive Joyful Attentive Training Intervention (NEJATI), and the hierarchical model of cognitive rehabilitation by Sohlberg and Mateer (Sohlberg & Mateer, 2017). Computer-based cognitive rehabilitation protocol was designed based on the Captain’s Log Computerized Cognitive Training System. The combined protocol of cognitive rehabilitation was constructed based on the Captain’s Log Computerized Cognitive Training System and Pars Cognitive Rehabilitation Package (Nejati, Shahidi & Helmi, 2016).

All three cognitive rehabilitation interventions were implemented in one hour once a week for 21 sessions. The contents of the sessions in three interventions included the following: memory (immediate or working memory; short-term memory; remote or long-term memory or the type of remembered information, including verbal; spatial; and motor skills), information processing speed (formulate an appropriate response, processing sentences and making sense of conversations, processing of visual information in a short distance, processing auditory, and the processing of incoming information), attention (selective attention, divided attention, alternating attention, and sustained attention), executive functions (planning and organizing sequencing, e.g. completing complex tasks, flexible thinking, motivation/drive, self-monitoring, problem-solving, self-correction, diminished abstract reasoning, poor decision making, & distractibility). In addition, these protocols comprised related psychoneurological skills, including linguistic functions and visual perceptual functions. The cognitive rehabilitative programs contained the main four steps. 1. Remediation: The retraining of disturbed functions. 2. Substitution: Reorganizing functions. 3. Accommodation: Promoting the use of preserved functions, and 4. Assimilation: Learning compensation strategies.

The study was reviewed and approved by the Institutional Review Board (IRB) in the Higher Education Center of Semnan University (Code 235/95/98). According to the ethical standards of human experimentation based on the Helsinki Declaration, the rights and welfare of study participants were protected during this study. The study was conducted concerning the professional ethics in research on humans, including confidentiality, the probability for leaving the study, and protection of the well-being of the study participants. In addition, informed written consent was obtained from all study participants at the study onset. Statistical analyses were performed in SPSS. The obtained data were analyzed by repeated-measures Multivariate Analysis of Variance (MANOVA).

## 3. Results

The study participants’ age ranged 18–45 years (Mean±SD: 29.65±7.47 y). The Mean±SD age of the computer-based intervention, the manual-based intervention, the combined intervention, the placebo, and the control groups were 30.166±7.20, 29.41±5.48, 27.83±8.49, 31.16±8.42, and 29.70±7.79 years, respectively. According to the educational level, the study participants were divided into 8 categories; elementary literacy (16.7%), high school literacy (16.7%), diploma (10%), associate degree (23.3%), undergraduate (20.3%), MSc degree (8.3%), and PhD. degree (5%). Moreover, 53.3% and 46.7% of the women were single and married, respectively. The studied women were diagnosed with MS for 2–7 years. Interferon-beta-1a (interferon beta 1-alpha) was the only drug prescribed for these patients. The Mean±SD values of working memory, selective attention, executive function, and information processing speed at the pre-test, post-test, and follow-up stages for all 5 groups are reported in Table 1.

**Table 1.** The participants’ descriptive statistics

**Intervention**		**Mean±SD**			

		**Working Memory**	**Selective Attention**	**Executive Function**	**Processing Speed**
Pre-test	Computer-based	19.3±2.74	12.25±2.18	13.42±1.57	1136.42±89.95
	Manual	19.08±2.71	11.92±1.17	13.25±1.42	1127.83±73.48
	Combined	19.41±2.64	12.08 ±1.44	13.33±1.92	1116.25±49.02
	Placebo	19.25±3.16	12.00±1.48	13.42±2.23	1119.75±72.01
	control	19.17±2.98	12.17±1.90	13.42 ±1.92	1125.58±52.04
Post-test	Computer-based	23.25±2.42	9.16 ±1.99	10.41±1.44	1033.59±35.80
	Manual	22.25±3.10	9.08±1.08	10.00±1.41	1051.00±61.48
	Combined	23.17±3.51	9.16±1.47	10. 17±1.94	1039.67±58.57
	Placebo	19.33±3.00	11.91±1.38	13.50±2.31	1120.75±74.74
	control	19.78±3.48	12.00±1.70	13.08±2.50	1124.17±87.30
Follow-up	Computer-based	22.25±2.00	9.58±1.88	11.08±1.31	1075.91±63.22
	Manual	20.67±2.80	9.91±0.90	10.83 ±1.40	1083.75±52.52
	combined	22.75±3.72	9.83±1.64	10.83±1.69	1055.83±60.42
	Placebo	19.33±2.64	11.91±1.24	13.50±2.40	1117.50±71.58
	control	19.59±4.03	12.00±1.41	13.16±2.40	1128.08±89.60

Repeated-measures MANOVA was applied to explored the effects of cognitive rehabilitation interventions assigned to working memory, selective attention, executive function, and information processing speed in three-time intervals. Initially, according to the statistical analysis assumptions, the obtained results suggested that the sphericity of all variables was rejected. To evaluate the Pillai’s effect, the size and corrected values of Huynh–Feldt were used. The assumed variances’ homogeneity was studied according to Levene’s test. Accordingly, we found that the assumption in 0.01 levels is true in all variants at pre-test, post-test, and follow-up stages.

The MANCOVA results indicated that group factor is not statistically significant (Pillai’s Trace=0.515, F_16,220_=2.033, P>0.001, Partial Eta-Square=0.1290), while the effects of time in Partial Eta-Square (Pillai’s Trace=0.884, F_8,48_=45.750, P<0.001, Eta-Square=0.884) and interaction of time in group Partial Eta were statistically significant (Pillai’s Trace=1.036, F_32,204_=2.228, P<0.001, Eta-Square=0.259). Moreover, the within-group MANCOVA results suggested that the effect of the time factor (Pillai’s trace=19.048, F_8,216_=19.048, P<0.001, Partial Eta-Square=0.414) and the time (Pillai’s Trace=0.824, F_32,440_=3.567, P<0.001, Partial Eta-Square=0.206) were significant. For the follow-up stage, the univariate analysis was used; the relevant results revealed that the main effect of time (pre-test, post-test, and follow-up) on working memory scores (F=76.776, P<0.001, Partial Eta-Square=0.583), selective attention (F=102.670, P<0.001, Partial Eta-Square=0.651), executive functions (F=141.540, P<0.001, Partial Eta-Square=0.720), and information processing speed (F=16.401, P<0.001, Partial Eta-Square=0.230) were significant.

The interaction with univariate follow-up tests was performed. The obtained data indicated that the interaction time in the working memory scores (F=10.924, P<0.001, Partial Eta-Square=0.443), selective attention (F=_15.012_, P<0.001, Partial Eta =0.522), and executive functions (F=20.976, P<0.001, Partial Eta-Square=0.604) were significant; however, the interaction effect of time in processing speed scores (F=2.923, P>0.001, Partial Eta-Square=0.175) was not significant. The findings on the comparison of differences between the control and placebo groups were not statistically significant (P>0.001).

**Figure 1.**
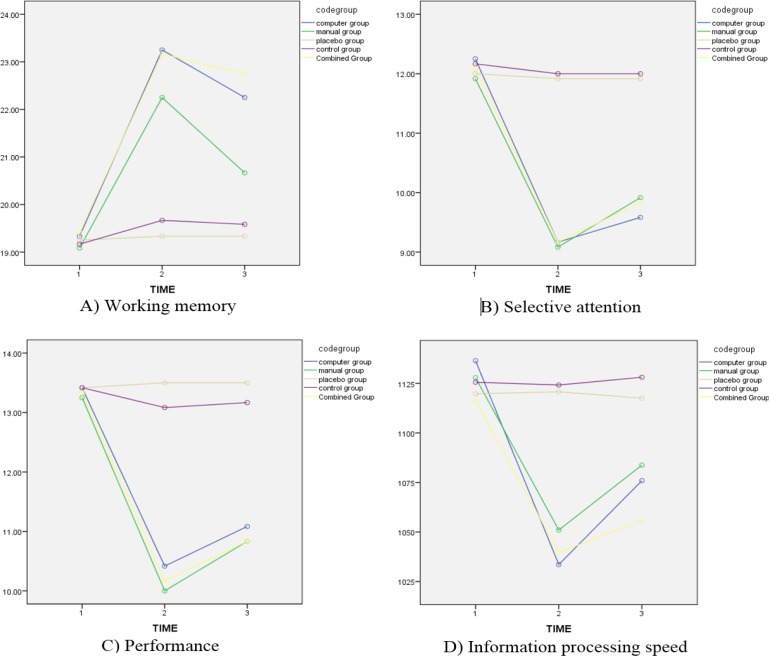
The effect of interaction time on working memory, selective attention, executive functions, and information processing speed

A paired comparison after the experience and adjustment was followed with the Bonferroni method (Table 2). The working memory capacity of computer-based and placebo groups was significantly different at the post-test (D=3.917, P<0.001). The comparison of the two groups revealed that the mean score of the placebo group was less than that of the computer-based group. This finding suggests that computer-based cognitive rehabilitation is effective in improving working memory (Figure 1A). In selective attention, two computer-based rehabilitation and placebo groups significantly differed at the post-test (D=2.750, P<0.05).

**Table 2.** A paired comparison of working memory, selective attention, executive functions, and processing speed

**Variables**	**Mean Difference of Experimental Group with Control Group**		

	**Pre-test**	**Post-test**	**Follow-up**
Working memory	250 ^1–2^, 0.083 ^1–3^, 0.167 ^1–4^, −0.083 ^1–5^, −1.67 ^2–3^, −0.83 ^2–4^, −0.333 ^2–5^, 0.083^3–4^, −0.0167 ^3–5^, −0.0250 ^4–5^	1.00 ^1–2^, 3.917 ^1–3***^, 3.583 ^1–4**^, 0.083 ^1–5^, 2.917 ^2–3*^, 2.583 ^2–4*^, −0.917 ^2–5^, −0.333 ^3–4^, −3.833 ^3–5**^, −3.500 ^4–5**^	1.583 ^1–2^, 2.917 ^1–3*^, 2.667 ^1–4*^, −0.500 ^1–5^, 1.333 ^2–3^, 1.083 ^2–4^, −2.083 ^2–5^, −0.250 ^3–4^, −3.417 ^3–5**^, 3.167 ^4–5**^
Selective attention	0.333 ^1–2^, 0.250 ^1–3^, 0.083 ^1–4^, 0.167 ^1–5^, −0.083 ^2–3^, −0.250 ^2–4^, −0.167 ^2–5^, −0.167 ^3–4^, −0.083 ^3–5^, 0.083 ^4–5^	0.083 ^1–2^, 2.750 ^1–3*^, 2.833 ^1–4*^, 0.000 ^1–5^, −0.083 ^2–3^, 2.917 ^2–4*^, −0.083 ^2–5^, −0.083 ^3–4^, 2.750 ^3–5*^, 2.833 ^4–5*^	−0.333 ^1–2^, −2.333 ^1–3*^, −2.417 ^1–4*^, −0.250 ^1–5^, −2.000 ^2–3*^, −2.083 ^2–4*^, 2.083 ^2–5*^, −0.083 ^3–4^, 2.083 ^3–5*^, 2.167 ^4–5*^
Executive functions	0.167 ^1–2^, 0.00 ^1–3^, 0.000 ^1–4^, 0.083 ^1–5^, −1.67 ^2–3^, −1.167 ^2–4^, −0.083 ^2–5^, 0.000 ^3–4^, 0.083 ^3–5^, 0.83 ^4–5^	0.417 ^1–2^, −3.083 ^1–3*^, 2.667 ^1–4*^, 0.250 ^1–5^, −3.500 ^2–3**^, −3.083 ^2–4*^, −0.167 ^2–5^, 0.417 ^3–4^, 3.333 ^3–5**^, 2.917 ^4–5*^	0.25 ^1–2^, −2.417 ^1–3*^, −2.083 ^1–4*^, 0.250 ^1–5^, −2.667 ^2–3*^, −2.333 ^2–4*^, 0.000 ^2–5^, 0.333 ^3–4^, 2.667 ^3–5*^, 2.333 ^4–5*^
Processing speed	8.58 ^1–2^, 16.667 ^1–3^, 10.833 ^1–4^, 20.167 ^1–5^, 8.083 ^2–3^, 2.250 ^2–4^, 11.583 ^2–5^, −5.833 ^3–4^, 3.500 ^3–5^, 9.333 ^4–5^	−17.417 ^1–2^, −87.167 ^1–3***^, −90.583 ^1–4***^, −6.083 ^1–5^, −69.750 ^2–3*^, −73.167 ^2–4*^, 11.333 ^2–5^, 3.417 ^3–4^, 81.083 ^3–5*^, 84.500 ^4–5**^	−7.833 ^1–2^, −41.583 ^1–3^, −52.167 ^1–4^, 20.083 ^1–5^, −33.750 ^2–3^, −44.333 ^2–4^, 27.917 ^2–5^, −10.583 ^3–4^, 61.667 ^3–5*^, 72.250 ^4–5*^

* P<0.05;

** P<0.01;

*** P<0.001

1–5: ^1^ Computer-based group; ^2^ Manual group; ^3^ Placebo group; ^4^ Control group; and ^5^ Combined group

Comparing these two groups highlighted that the mean score of the placebo group was lower than that of the computer-based rehabilitation; thus, the intervention was effective in improving selective attention. In selective attention, computer-based rehabilitation and control groups significantly differed at the post-test (D=3.583, P<0.01). The selective attention between manual and computer-based rehabilitation was significantly different at the post-test (D=2.917, P<0.05). Moreover, the selective attention between computer-based rehabilitation and control groups was significantly different at the post-test (D=2.833, P<0.05); accordingly, the intervention was effective on selective attention at the follow-up stage. The mean difference between computer-based and placebo groups (D=-2.833, P<0.05), computer-based and control groups (D=-2.417, P<0.05), manual and placebo groups (D=-2.000, P<0.05), as well as manual and control groups (D=-2.083, P<0.05) were statistically significant.

At post-test, all comparisons between the three experimental groups and the control and placebo groups were statistically significant (P<0.05). In addition, at follow-up, most of these comparisons were statistically significant (P<0.05).

The working memory in computer-based and placebo groups (D=3.917, P<0.001), computer-based and control groups (D=3.583, P<0.01), manual-based and placebo groups (D=2.917, P<0.05), manual-based and control groups (D=2.583, P<0.05), combined and placebo groups (D=-3.833, P<0.01), and combined and control groups (D=3.500, P<0.01) were significantly different at post-test. These data suggest the effectiveness of the intervention in all three computer-based, manual-based, and combined cognitive rehabilitation on working memory (Figure 1A).

The effects of the three rehabilitation methods (computer-based, manual-based, and combined) with the control (D=2.833, P<0.05; D=2.917, P<0.05; D=2.833, P<0.05) and computer-based and combined intervention with placebo group (D=2.750, P<0.05; D=2.750) was effective on improving selective attention at post-test (Figure 1B).

The executive functions of computer-based and placebo groups (D=-3.083, P<0.05), computer-based and control groups (D=−2.667, P<0.05), manual-based and control groups (D=−3.083, P<0.05), combined and placebo groups (D=3.333, P<0.01), and combined and control groups (D=2.917, P<0.05) were significantly different at post-test. The executive function in computer-based and placebo groups (D=−2.417, P<0.05), manual-based and placebo groups (D=−2.667, P<0.05), manual-based and control groups (D=−2.333, P<0.05), combined and placebo groups (D=2.667, P<0.05), and combined and control groups (D=2.333, P<0.05) were significantly different at follow-up. Thus, all three (computer-based, manual-based, and combined) methods of rehabilitation were effective in executive functions (Figure 1C). Information processing speed in computer-based and placebo groups (D=−87.167, P<0.001), combined and placebo groups (D=81.083, P<0.05), and combined and control groups (D=84.500, P<0.01) were significantly different at post-test. Thus, it can be suggested that the combined effect of the two methods can improve the information processing speed (Figure 1D).

## 4. Discussion

Of the obtained data suggested no significant difference between the implemented cognitive rehabilitation (computer-based, manual-based, and combined); however, they highlighted the effectiveness of each approach on working memory, selective attention, executive function, and information processing speed, compared to the placebo and control groups.

The collected results were consistent with those of the computer-based cognitive rehabilitation developed by Lincoln et al.; they could improve attention and memory in MS patients (Lincoln et al., 2015). In addition, a clinical trial conducted by Cerasa et al. (2013) for improving attention deficits suggested progressed intentional functions in MS patients (Cerasa et al., 2013). Vogt et al. (2009) in a computer-based training about executive functions, including working memory in patients with MS reported the relative effectiveness of computer rehabilitative program on processing speed and working memory in 30 patients and 20 healthy controls in 45-minute sessions per week (Vogt et al., 2009). Additionally, indicated intensive cognitive rehabilitation, including, memory techniques, enhanced executive functions, especially memory and recall. Their work could markedly enhance the ability of patients with cognitive impairment, compared with the controls. In the randomized clinical trial, new techniques were used to improve learning and memory performance in MS patients. They concluded that memory impairment in MS could be effectively treated through a rehabilitation program memory using text and images.

Bonavita et al. (2015) implemented a Computer-aided cognitive rehabilitation program to improve cognitive performances; they reported significant improvement in the training group compared to the controls with cognitive abilities (Bonavita et al., 2015). Moreover, they argued that personal cognitive training is a practical and valuable procedure for improving cognitive abilities and reducing neurocognitive signs in RRMS (Bonavita et al., 2015). Pusswald et al. documented that the computer-based neuropsychological rehabilitation can significantly improve executive functions, including processing speed, cognitive flexibility, visual and verbal declarative memory performance, prefrontal cortex efficacy, administrative skills, and memory in this group (Pusswald, Mildner, Zebenholzer, Auff, & Lehrner, 2014). Moreover, the obtained results are consistent with those of Mäntynen et al. (2014); they applied a computer-based neuropsychological rehabilitation and concluded that this intervention reduces cognitive deficits among MS patients (Mäntynen et al., 2014).

As previously noted, this study detected a statistically significant difference between the computer-based, manual-based, and combined cognitive rehabilitation groups, and the control and placebo groups in terms of the pre-test, post-test, and follow-up scores. According to these results, computer-based, manual-based, and combined cognitive rehabilitation were effective in working memory, selective attention, executive functions, and information processing speed. As Bonavita et al. (2015) noted, computer-aided cognitive rehabilitation and training affect the neuropsychological mechanisms and might improve memory, attention, and executive functions (Bonavita et al., 2015). The computer-based cognitive rehabilitation, manual-based cognitive rehabilitation, or a combination of computer-based and manual-based cognitive rehabilitation decelerate the loss of cognitive deficits among MS patients. This because of cognitive plasticity toward cognitive training in these patients. Accordingly, planning strategies and treatment agenda to recover and prevent cognitive impairments is important for MS patients. In addition, cognitive functions in MS may be remediated due to cognitive rehabilitation; computer- or manual-based interventions impact the prefrontal and frontal functions, including cognitive problems.

Furthermore, this treatment is accompanied by cognitive remediation and the reinforcement of adaptive cognitive abilities. Mattioli et al. suggested that new technologies for cognitive enhancement, including computerized cognitive training plans, are objective, convenient, and cost-benefit for administration by trainers and trainees; as tailored interventions, they have effectively enhanced cognitive functions. Pusswald et al. argued that cognitive rehabilitation is especially effective when patients have moderate to severe memory impairments (Pusswald et al., 2014). Besides, concerning the lack of significant differences among all interventions, cognitive rehabilitation is a tailored intervention; it has effectiveness and efficacy unrelated to administration format and applied in the background of multidisciplinary interventions.

To our knowledge, this was the first quasi-experimental study with a randomized clinical trial design to compare the effectiveness of computer-based, manual-based, and combined cognitive rehabilitation on improving the cognitive functions of patients with RRMS. The cognitive rehabilitation effects vary in patients with diverse neuropsychological profile and the distinctive severities of cognitive impairments; thus, different rehabilitation programs, including computer-based, manual-based, or combination cognitive rehabilitation may be required in MS. The benefits of computer-based cognitive rehabilitation include convenient access to the intervention, low cost in a real-world application, and powerful intervention adherence. These main reinforcements provide a greater tendency to applying computer-based cognitive rehabilitation in MS management in the future.

Implementing the tailored and curative cognitive rehabilitative programs in cognitive impairments at the onset or earlier phases of MS before the deterioration of patient’ status is important. Cognitive rehabilitation manuals are encouraging in this respect. These results share in bringing about to further progression of MS rehabilitation programs. They will also provide information to health-care providers for providing tailored cognitive rehabilitation services for patients with MS. Consequentially, this study overcame the shortcomings of numerous studies, including no unrepresentative consequences and passive controls with the placebo group. As study strength, patients with specific clinical features (RRMS) enabled the researchers to provide a tailored intervention in this study.

The project was based on the development of a contraceptive method, and noticeable impacts might not be achieved in a short time. Therefore, performing long-term follow-ups are required. In this regard, an 18-month follow-up seems adequate for this program; however, it can be extended if a longer follow-up for referring the patient to association and hospital is needed. Another limitation of this study was the inability to apply the techniques on patients in the advanced stages. The study only focused on some cognitive functions (working memory, selective attention, executive functions, and information processing speed); thus, generalizing it to other cognitive functions might not be correct.

Furthermore, this study was performed only on female patients with RRMS. Thus, it is suggested to implement it on broader scales and men as well as other MS types (e.g. primary and secondary progressive). Due to irreversible cognitive damage in the advanced stages of MS, it is also recommended to continue the cognitive rehabilitation programs immediately after the diagnosis along with pharmacotherapy. Moreover, the present study disregarded matching the socioeconomic status of the study groups. The statistical sample was small, comparative to the population heterogeneity; the obtained results’ generalizability is restricted. However, evidence provided no socioeconomic differences in these interventions affect the interpretation of the results. Another limitation was that fatigue and depression (due to controlling them) were not included in this study. These symptoms frequently occur among MS patients and might lead to cognitive impairments. It is suggested that the psychopathological comorbidity be included in future studies.

## 5. Conclusion

Cognitive rehabilitation was effective in improving cognitive functions in patients with RRMS. Thus, these interventions are recommended for application, along with other treatment protocols to treat RRMS in clinical settings.
